# Evaluation of Rapid Antigen Tests Using Nasal Samples to Diagnose SARS-CoV-2 in Symptomatic Patients

**DOI:** 10.3389/fpubh.2021.728969

**Published:** 2022-01-14

**Authors:** Manaf Alqahtani, Abdulkarim Abdulrahman, Fathi Mustafa, Abdulla I. Alawadhi, Batool Alalawi, Saad I. Mallah

**Affiliations:** ^1^National Taskforce for Combating the Coronavirus (COVID-19), Sanabis, Bahrain; ^2^Royal College of Surgeons in Ireland-Bahrain, Al Muharraq, Bahrain; ^3^Bahrain Defence Force Hospital, Riffa, Bahrain; ^4^Mohammed Bin Khalifa Cardiac Centre, Riffa, Bahrain; ^5^Ministry of Health, Sanabis, Bahrain

**Keywords:** SARS-CoV-2 (2019-nCoV), nasopharyngeal swabs, nasal swab, rapid antigen detection test, RT-PCR, viral diagnostic, COVID-19

## Abstract

**Introduction:**

The best way to mitigate an outbreak besides mass vaccination is via early detection and isolation of infected cases. As such, a rapid, cost-effective test for the early detection of COVID-19 is required.

**Methods:**

The study included 4,183 mildly symptomatic patients. A nasal and nasopharyngeal sample obtained from each patient was analyzed to determine the diagnostic ability of the rapid antigen detection test (RADT, nasal swab) in comparison with the current gold-standard (RT-PCR, nasopharyngeal swab).

**Results:**

The calculated sensitivity and specificity of the RADT was 82.1 and 99.1%, respectively. Kappa's coefficient of agreement between the RADT and RT-PCR was 0.859 (*p* < 0.001). Stratified analysis showed that the sensitivity of the RADT improved significantly when lowering the cut-off RT-PCR Ct value to 24.

**Conclusion:**

Our study's results support the potential use of nasal swab RADT as a screening tool in mildly symptomatic patients, especially in patients with higher viral loads.

## Introduction

Since December 2019, the number of Coronavirus disease 2019 (COVID-19) confirmed cases has been rising rapidly despite the efforts to limit its spread (1, 2). The World Health Organization (WHO) declared COVID-19 to be a pandemic on March 12, 2020 (2). To date, the total number of cases worldwide has exceeded 120 million, with over 2.5 million deaths (3). The National COVID-19 Taskforce in Bahrain has been working diligently to confine this disease's spread since the start of the pandemic. Bahrain has had more than 140,000 COVID-19 cases, afflicting about 8% of the population (4).

One of the most effective ways to mitigate a viral outbreak in the absence of population-wide vaccination is the efficient detection of cases early enough to take the necessary precautions that could halt its spread to contacts and allow for the adequate management of high-risk patients. However, this is difficult to achieve in the absence of a readily available, rapid, and cost-effective test with sufficiently high specificity and sensitivity for early detection of COVID-19 infected patients in the general population (5–8).

Until now, nasopharyngeal Real-Time Polymerase Chain Reaction (RT-PCR) is the gold standard diagnostic test for COVID-19 (5–8). RT-PCR has multiple limitations, including delayed availability of results and the need for specialized laboratory equipment as well as specialized technicians (1, 5, 6, 8). As a result, the number of tests performed per day is restricted by these limitations, risking delaying the appropriate management of positive cases. Therefore, other diagnostic techniques are needed to limit the virus's spread and effectively monitor the degree of COVID-19 infection in the population (1, 5, 6, 8). Current literature explores the possibility of using point-of-care rapid antigen tests as a cost-effective and simple modality that has been used effectively with other viruses such as Influenza and respiratory syncytial virus (RSV) (9). However, the studies report an overall low sensitivity and high specificity compared to RT-PCR (1, 5, 6, 8).

Our study explores nasal swabs' diagnostic performance as they do not require a skilled professional, are less time consuming, and cause less discomfort. Furthermore, nasal swabs—which are routinely used in microbiology labs with no risk of supply disruption—have been validated as an alternative procedure to collect nasal secretions, with nearly equivalent detection abilities to nasopharyngeal swabs (10). Nasopharyngeal swabs however are the reference sampling method for the detection of SARS-COV-2 as per the World Health Organization (11). We aim to demonstrate the efficacy of nasal antigen tests in mildly symptomatic cases. This would provide a simple, reliable test that might eliminate negative cases with a certain level of confidence. Implementation of such tests will reduce the workload on healthcare professionals and institutions, as these tests can be done at clinics or home and facilitate reopening and relaxing nationwide restrictions.

## Objective

To determine the nasal swab antigen test's accuracy in detecting SARS-COV-2 compared to nasopharyngeal RT-PCR in mildly symptomatic individuals.

## Methods

### Study Population

The study involved 4,183 mild symptomatic individuals. Definition of “mildly symptomatic” individuals followed Bahrain's protocol (12). It included fever (<38°C), loss of taste or smell, flu-like symptoms, sore throat, gastrointestinal symptoms, myalgia, and fatigue. The study participants were referred to the national testing center's symptomatic hall at the Bahrain International Exhibition and Convention Center.

### Setting

All testing was conducted in the symptomatic hall in the National Testing Centre at the Exhibition Centre in Manama, Bahrain.

### Study Design

We conducted a cross-sectional study to determine the diagnostic performance of the rapid antigen test compared to RT-PCR. Two swabs were taken from each individual, one nasal swab for the antigen test and one nasopharyngeal swab for the RT-PCR. For rapid antigen test, Abbott panbio COVID-19 antigen rapid test device (Abbott Rapid Diagnostic Jena GmbH, Jena, Germany) to detect SARS-CoV-2 nucleocapsid protein was used. The contained membrane strip is pre-coated with immobilized anti-SARS-CoV-2 antibody on the test line and mouse monoclonal anti-chicken IgY on the control line (13). The nasopharyngeal samples for RT-PCR were transferred to a viral transport media immediately after collection and transported to a COVID-19 laboratory for testing. The RT-PCR test was conducted using Thermo Fisher Scientific (Waltham, MA) TaqPath 1-Step RT-qPCR Master Mix, CG on the Applied Biosystems (Foster City, CA) 7500 Fast Dx RealTime PCR Instrument. The assay used followed the WHO protocol and targeted the E gene. If the E gene was detected, the sample was then confirmed by RdRP and N genes (14). The E gene Ct value was reported and used in this study. Ct values >40 were considered negative. Positive (virus-like particles of SARS-CoV-2 and RNase P) and negative (RNase-free Water) controls were included for quality control purposes.

### Sample Collection

All samples were collected by a trained healthcare professional in the national testing center. The nasal samples were collected using the nasopharyngeal swab provided with the RADT kit from both nostrils. Based on the CDC guidelines, the patient's head was tilted back by 70°. The swab was inserted approximately 2 cm into the nostril while gently rotating it, rolling it several times before removing it. The swab tip was placed in the buffer fluid inside the extraction tube, with 5-drops of extracted specimen dispensed onto the specimen well (S) on the device. Results were read after 15 min.

The nasopharyngeal samples used for RT-PCR were collected through both nostrils from the nasopharynx using a nasopharyngeal swab. The nasopharyngeal swab was inserted into the nostril parallel to the palate until resistance was encountered, or the depth was equivalent to the distance of the nose from the ear. The swab was rolled and rubbed gently, left in place for multiple seconds, then removed slowly while rotating it and placed into the transport tube (15).

### Participants

- Inclusion criteria:
  ° Suspected COVID-19 cases with mild symptoms [defined by Bahrain's protocol (12)] presenting to the testing center.
- Exclusion criteria:
  ° Suspected cases with severe symptoms
  ° Any asymptomatic suspected case

### Data Handling and Statistical Analysis

Antigen test results and RT-PCR result with the corresponding Ct value were collected for all mildly symptomatic cases. The antigen's diagnostic performance was assessed using sensitivity, specificity, positive predictive value, negative predictive value, and respective 95% Confidence interval. Agreement between nasopharyngeal RT-PCR and nasal antigen tests was assessed using kappa coefficient of agreement. The Ct value of identified and missed cases by antigen tests were summarized using median and interquartile range. Ct Value of identified and missed cases were compared using a two sample *t*-test. All *p*-values were two-sided, and *P* < 0.05 was considered significant. Data collection was performed through a live google sheet and extracted to Microsoft Excel 2016. Statistical analysis was performed using STATA (16).

### Ethical Considerations

Ethical and research approval was obtained from the National COVID-19 Research and Ethics Committee (approval code: CRT-COVID-2020-088). All methods and analysis of data were approved by the National COVID-19 Research and Ethics Committee and carried out according to the local guideline and ethical guidelines of the Declaration of Helsinki 1975. Written Informed consent was waived by the Research and Ethical Committee for this study due to the absence of any patient identifying information.

## Results

A total of 4,183 mild symptomatic cases were tested by RT-PCR (using a nasopharyngeal sample) and by antigen test (using a nasal sample). 56.5% of the cases were males, and 43.5% were females. The mean age of the tested population was 30.9 years (± 14.5 years). Days from symptom onset ranged from 0 to 14 with a median of 2 (IQR 1–3). Table 1 summarizes the demographics of the tested cohort. 17.5% (733/4,183) of the population tested positive by RT-PCR; no equivocal results were reported. Using the antigen test, 15.1% were positive, while the remaining tested negative, and none of the tests were equivocal.

**Table 1:** Demographics and clinical features of studied sample.

**Variables**	***N***	
Age in years–Mean ± SD	4,183	30.9 ± 14.5
Male–no. (%)	4,183	2,365 (56.5%)
Symptoms Onset in days–Median (IQR)	1,301	2 (1–3)
Prevalence–no. (%)	4,183	733 (17.5%)
Ct Value of PCR positive cases–Median (IQR)	530	22 (20–24.5)

Out of the 733 confirmed RT-PCR cases, the antigen test accurately diagnosed 632 cases (82.1%). One hundred and thirty five cases were falsely negative by the antigen test, and 30 cases were reported as false positive. Table 2 is a contingency table showing the RT-PCR and Antigen test results. Using nasopharyngeal RT-PCR as the gold standard test for diagnosis of SARS-CoV-2, the rapid antigen test showed a sensitivity of 82.1% (95% CI 79.2–84.8%) and a specificity of 99.1% (95% CI 98.8–99.4%). With the prevalence of COVID-19 being 17.5% within the tested population, the antigen test had a positive predictive value (PPV) of 95.3% and a negative predictive value (NPV) of 96.3%. Agreement analysis between the nasopharyngeal RT-PCR and the nasal antigen test showed 85.9% observed agreement (κ coefficient = 0.859, *p* < 0.001). Table 3 summarizes the diagnostic performance of the antigen test.

**Table 2:** 2 × 2 table showing the PCR and Antigen test results.

	**PCR +**	**PCR –ve**	**Total antigen results**
Antigen test positive	602 True positive	30 False positive	632
Antigen test negative	131 False negative	3,420 True negative	3,551
Total PCR results	733	3,450	Total cases: 4,183

**Table 3:** Assessment of the diagnostic accuracy of the antigen test.

	**Value**		**95% CI**	
Prevalence	17.5%	16%		18.7%
Sensitivity	82.1%	79.2%		84.8%
Specificity	99.1%	98.8%		99.4%
Positive predicted value	95.3%	93.3%		96.8%
Negative predicted value	96.3%	95.6%		96.9%
Positive likelihood ratio	94.4	66		135
Negative likelihood ratio	0.18	0.154		0.211
False discovery rate	3.8%	3.3%		4.4%
Kappa coefficient^**^	85.9%	83.8%		88%

** *The p-value of the kappa coefficient p < 0.001*.

**Figure 1:**
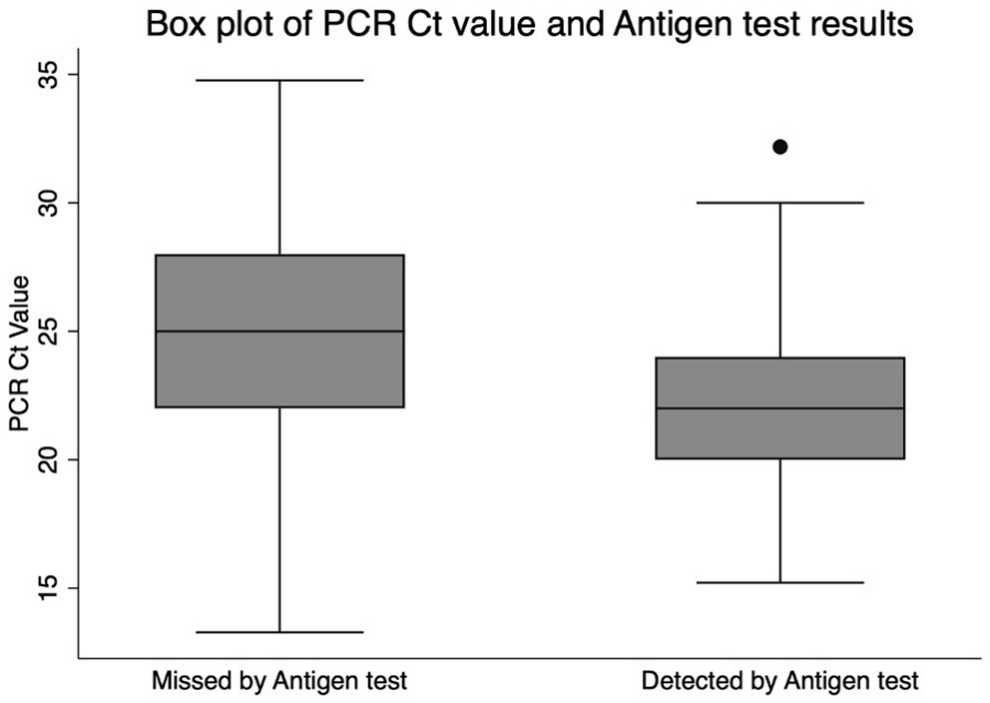
Box plot of PCR Ct value and Antigen test result.

Confirmed cases had a median Ct value of 22 (IQR 20–24.1). Cases detected by the antigen test had a median Ct value of 22 (IQR 2–24) and a mean of 22.1 (95% CI 21.9–22.4). Cases missed by the antigen test had a median Ct value of 25 (IQR 22–28) and a mean of 25.1 (95% CI 24.3–25.8). The mean Ct value difference between the false negative and the true positive cases was statistically significant (t-score 9.2, *p* < 0.001). The median Ct values and their corresponding interquartile ranges are shown in Figure 1.

To control for time since symptom onset as a confounder, we performed a stratified analysis to assess the significance of time since onset of symptoms on the antigen test's diagnostic performance. Cases with symptom onset within 5 days showed a modest improvement in the diagnostic performance with a sensitivity of 82.4%, specificity of 99.3%, and a Kappa coefficient of 0.865. This was almost similar to cases with symptom onset within 7 days, as shown in Table 4. Additionally, a secondary analysis was conducted after excluding cases with Ct values more than or equal to 30 and Ct more than 24. The sensitivity increased to 84.5 and 87.9%, respectively. In contrast, specificity for both cutoff Ct values was 99.1%. Moreover, after excluding cases with Ct value > 24 and restricting symptoms onset to within 5 and 7 days, there was a significant increase in sensitivities up to 89.5 and 89.3%, respectively.

**Table 4:** The effect of symptoms onset time and Ct values on the diagnostic performance.

**No**.	**Model**	***N***	**Prevalence**	**Sensitivity**	**Specificity**	**Negative predicted value**	**Positive predicted value**	**Kappa (*p* <0.001)**
1	Symptom onset within 7 days	1,290	20% (18–22.8%)	82.6% (77.5–87%)	99.3% (98.6–99.7%)	95.7% (94.3–96.8%)	96.9% (93.7–98.7%)	86.6% (83.1–90.1%)
2	Symptom onset within 5 days	1,252	20% (18–22.8%)	82.4% (77.2–86.9%)	99.3% (98.6–99.7%)	95.6% (94.2–96.8%)	96.8% (93.5–98.7%)	86.5% (82.9–90.1%)
3	Excluding cases with Ct ≥ 30	4,148	17% (16–18.1%)	84.5% (81.6–87.1%)	99.1% (98.8–99.4%)	96.9% (96.3–97.5%)	95.2% (93.2–96.7%)	87.5% (85.5–89.6%)
4	Excluding cases with Ct > 24	3,996	14% (13–15%)	87.9% (84.9–90.5%)	99.1% (98.8–99.4%)	98.1% (97.6–98.5%)	94.2% (91.8–96.1%)	89.5% (87.5–91.6%)
5	Symptom onset within 7 days and Excluding cases with Ct ≥ 30	1,274	20% (17–21.8%)	86.3% (81.4–90.4%)	99.3% (98.6–99.7%)	96.8% (95.5–97.8%)	96.8% (93.6–98.7%)	89.3% (86.1–92.5%)
6	Symptom onset within 7 days and Excluding cases with Ct > 24	1,220	16% (14–18.3%)	89.3% (84.2–93.3%)	99.3% (98.6– 99.7%)	98% (96.9–98.7%)	96.2% (92.3–98.4%)	91.3% (88.1–94.5%)
7	Symptom onset within 5 days and Excluding cases with Ct > 30	1,236	19% (17–21.8%)	86.3% (81.3–90.4%)	99.3% (98.6–99.7%)	96.8% (95.5–97.8%)	96.7% (93.4–98.7%)	89.3% (86.0–92.5%)
8	Symptom onset within 5 days and Excluding cases with Ct > 24	1,184	16% (14–18.3%)	89.5% (84.2–93.5%)	99.3% (98.6–99.7%)	98% (96.9–98.8%)	96% (92–98.4%)	91.3% (88.1–94.5%)

As a follow-up, cases that tested negative by the antigen test and tested positive by the RT-PCR were asked to repeat the antigen test within 72 h. Nineteen out of 135 responded, and 73.7% were positive on the repeated antigen test. Three of the 30 cases tested positive by the antigen test but negative by RT-PCR were tested again within 72 h. One case tested negative, while two remained positive and tested positive by repeat RT-PCR.

## Discussion

The RT-PCR has been a very accurate test to diagnose all kinds of infectious diseases. It provides results faster than cultures, and its use for early diagnosis by infectious disease specialists has been very popular (17). During the pandemic, the RT-PCR test was the only accurate test available to diagnose infected individuals (18). RT-PCR is a very sensitive test for SARS-CoV-2, and this sensitivity had improved within a few months into the pandemic. Some RT-PCR machines detect down to ten viral RNA copies μl^−1^ (19). Despite the RT-PCR test's high sensitivity, it has multiple limitations that hold back the efforts in battling this pandemic with reopening plans in motion worldwide. Numerous studies showed that RT-PCR was sometimes positive in patients with a corresponding negative culture test for SARS-CoV-2, which indicates that these patients were not infectious (8, 20). This has led to the isolation of people who are noninfectious and halted reopening measures. Another limitation is that it requires healthcare professionals to collect the swab and specialized labs and specialists to analyze and interpret the result (5, 6).

As the pandemic necessitated mass testing, the turnaround time extended and required average 2–3 days in many countries (21, 22). This time limitation has kept Bahrain under-armed when fighting the pandemic. One of the main steps to mitigate this outbreak's spread is to have an accurate test that will detect infectious individuals who pose a public health risk and report results quickly. The test should also be easy to perform by the general population and repeat multiple times whenever necessary. This will reduce the workload on healthcare professionals as well as smoothen the reopening process. The use of the nasopharyngeal swab is a limiting factor in terms of ease and frequency of testing because it is invasive, uncomfortable, and aerosolizing (23). For similar reasons, the Centers for Disease Control and Prevention (CDC) permitted self-sampling via nasal swabs to compensate for the shortage of healthcare workers and the escalation of COVID 19 cases (24). Furthermore, the CDC, along with several studies, have illustrated that supervised nasal swabs were quite as effective as nasopharyngeal swabs in detecting SARS-CoV-2 (17, 25).

The antigen test used in our study demonstrates that it can be a good test to be used in this context. The nasal antigen test had a significant agreement correlation of 85% with the nasopharyngeal RT-PCR in the studied population. The mild to moderate symptomatic population represents most COVID-19 cases; 81% as reported by a Chinese cohort (26). Hence targeting this population was our priority when investigating a newer test like RADT. Additionally, we excluded asymptomatic patients as the scope of this study focused on the appropriateness of RADT and the factors that might impact its performance in symptomatic individuals. In cases where patients present with severe disease, the RT-PCR test should continue to be used as having a definite result is necessary.

Our study's rapid antigen detection test (RADT) had a very high specificity of 99.3%. The test also had a high predictive value within a population with an 18% prevalence of COVID-19. The sensitivity of the test was 82.1% when compared to the RT-PCR test. Despite the antigen test having lower sensitivity, it was done using a nasal and not a nasopharyngeal sample. Moreover, RT-PCR's diagnostic accuracy can never be fairly compared to the rapid point of care antigen test as the detection method is different.

Our study's findings regarding the rapid antigen test's diagnostic performance match the data in the current literature to a certain extent. For example, a review of nine studies involving 7 different brands of rapid antigen tests reported that all studies demonstrate very high specificities. The pooled specificity was 99% (95% CI 98–100%), similar to our test's specificity (99.2%). However, the reported pooled sensitivity was 49% (95% CI 28–70), much lower than our test's sensitivity (81.3%). However, a wide range of sensitivities was reported across the studies, ranging from 0 to 94% (27). Few high-quality studies showed that some tests, such as the Bioeasy 2019-nCov Ag Fluorescence Rapid Test Kit, had a relatively high pooled sensitivity of 82.3%, which is close to our test's sensitivity (28).

Multiple studies reported either low sensitivities, such as 30 and 50%, or low Cohen's kappa coefficient of agreement, while in our study, the reported sensitivity was 81.3% (5, 7, 29). All of the studies mentioned above were using different commercial antigen tests and different swabs (nasopharyngeal swabs and nasal swabs). Moreover, most of the studies did not specify the severity of symptoms within the study population. The studies that reported very high sensitivities of the rapid antigen test usually involved patients who were either in the emergency department or hospitalized. Such patients are usually more symptomatic, hence have a higher viral load. As a result, the reported sensitivities were higher compared to patients with milder symptoms (30–33).

Our study's cases missed by the antigen test had higher Ct Value than those detected by the antigen test. The mean Ct value for the missed cases was 25.1. Bullard et al. described that viral cultures fail when the time from symptom onset exceeds 8 days and/or the Ct value exceeded 24 (34). When we excluded cases above the Ct value of 24, the sensitivity improved to 87.9% with an agreement rate (kappa coefficient) of 89.2 between nasal antigen test and nasopharyngeal RT-PCR. The accuracy improved further when symptom onset was restricted to 7 days and cases above Ct of 24 were excluded. The agreement coefficient reached 91.3% and sensitivity reached 89.3% without affecting specificity. This finding was also reported by Bayona et. al in a meta-analysis conducted on multiple RADT, which demonstrated that the sensitivity of the RADT was higher when performed in patients early in the disease (0–7 days) compared to tests performed late in the disease (8–14 days). The study also showed that the reduction of the Ct value from ≤ 40 to ≤ 30 increased the sensitivity of the rapid antigen test from 68 to 98%. One of the studies included in the meta-analysis showed that the sensitivity improved to 82.2% in patients with higher viral loads (Ct value < 25) (7). In addition, the median Ct value of antigen test negative cases was higher and significantly different from positive cases (7). The antigen test's sensitivity significantly improves when cases with high Ct values (30–40) were removed from the analysis (27, 35), and this was also proven by our study. Rapid antigen tests were sensitive enough to detect cases of early symptomatic cases with a high viral load, which likely account for a significant proportion of transmissions. This early detection can enable rapid isolation of cases with rapid initiation of contact tracing (36).

To implement the use of point of care (POC) rapid antigen testing in clinics as well as by the public, we need to improve the efficacy of the test by testing and implementing a scheme that would limit the number of false-negative cases, especially in symptomatic patients. We believe that increasing the frequency of the test can improve its diagnostic accuracy. As seen in the sample of 22 patients who had false results by the antigen test in our study, the repeated test showed accurate results in 77% of the repeated test. Additionally, since cases with higher viral loads are better detected by the RADT, repeating the test after a few days to allow the viral load to increase may be considered. Similarly, as per the European CDC, repeating the test 2–4 days after a confirmed contact tests negative would decrease the chances of a false negative (36). We have proposed an algorithm that can further improve the diagnostic accuracy of the test in symptomatic patients:

1. The RADT must not be used if more than 7 days have passed since symptoms onset or if the patient has severe symptoms.
2. If the RADT was negative, the individual should self-isolate until a true-negative is confirmed by RT-PCR within 24 h. In cases where an RT-PCR test may not be feasible, a repeat RADT may be considered after 24–48 h.
3. If the RADT was positive, the individual must self-isolate until an RT-PCR is performed soon after to confirm the diagnosis.

This algorithm however should be examined to understand the value of repeating an antigen test in those cases. Moreover, the time frame to repeat the RADT has to be investigated, to better identify an appropriate time range to improve the efficacy of the scheme. It is important to note that this algorithm for symptomatic patients leans on the side of caution, as RT-PCR remains the gold standard for COVID-19 diagnosis. The conduction of a RADT test allows for rapid at-home testing, eliminating the risk of transmission posed when a symptomatic individual visits a healthcare facility to get an RT-PCR test. It would allow for self-isolation while providing the medical taskforce more time to act and arrange an RT-PCR test for the patient. As such, the definition of mild symptoms must be clearly understood by the public. If a person had severe symptoms or was a high-risk individual (close contact), they must perform an RT-PCR first as these are higher risk populations that require a more accurate diagnosis. Antigen tests can thus be used in addition to RT-PCR as part of the testing strategies for COVID-19. The use of antigen tests can potentially decrease the use of RT-PCR tests.

In the case of mass screening of asymptomatic individuals with no known exposure (low pre-test probability), a negative RADT test, especially one followed by another negative RADT test a few days later, may suffice for a confirmation. In such cases, following every negative RADT test with a confirmatory RT-PCR would be counterproductive to the aim of easy and rapid mass screening. This recommendation is also in accordance with the US CDC's guidelines for a negative RADT in a population with a low pre-test probability (asymptomatic and no known exposure) (37).

Given the high specificity shown by the RADT, we believe that it can be adequately used in asymptomatic individuals who are not close contacts. The RADT can be used in different settings (gatherings, schools, and workplaces) to conduct frequent monitoring of the population and help in identifying cases early to prevent an outbreak. However, its diagnostic accuracy in these settings has to be examined to determine its efficacy.

The study has several strengths. The large sample size and the comparison of nasal swabs tested by RADT to Nasopharyngeal samples tested by RT-PCR are the two main unique strengths of this study. Moreover, the use of a single large testing center allowed standardization and increased quality in sample and data collection. All nasopharyngeal samples were transported and tested in a single lab using the same kits and machines and hence standardizing the results and Ct values. Our study provides novel data from the Eastern Mediterranean Health Region, contributing to the reproducibility and generalizability of current and future studies, and any upcoming meta-analyses.

The study has its limitations, the nasal sample was collected using nasopharyngeal swabs. Nasopharyngeal swabs are flexible and smaller and hence are more difficult to collect nasal samples. Therefore, this could have underestimated the results of the study. Furthermore, although it is of great value to compare nasopharyngeal RT-PCR to nasal RADT, the comparison of antigen tests to RT-PCR cannot be fairly deduced as the method differs; ideally, nasal swabs for both diagnostic modalities would be tested for Ct value accuracy. Additionally, both the RADT and RT-PCR tests were conducted by healthcare professionals, which was done to ensure standardization and limit bias; however, as a result, the demonstrated diagnostic strength of nasal RADT tests cannot be transferred to its use by unskilled professionals with full confidence. It remains a non-technical skill however, and as such the data should not defer in any significant way. Moreover, the participants' clinical symptoms were not collected, and there were significant amounts of missing data on time from symptom onset. This had led to a decrease in sample size when testing different models based on the restriction of time from symptom onset. This can either under or overestimate the results for the restricted models. Only a small number of cases agreed to have a repeated test after a discrepancy in RT-PCR and RADT results. The timing of the repeat test ranged from 24 to 72 h and wasn't standardized due to logistical difficulties.

## Conclusion

The sensitivity of rapid antigen tests is affected by numerous factors including the viral load, the onset of symptoms, route of sample collection, and the circumstances in which it was used. The results of the diagnostic assessment of nasal swabs in the RADT used in our study are promising regarding the potential benefit of using them as a screening tool in mildly symptomatic patients. The diagnostic ability was especially high in cases with a high viral load. Further investigations ought to be performed to test the algorithms/protocols of repeated testing using RADT to further improve its diagnostic ability. More research is required to assess the ability of the RADT to screen large populations with low disease prevalence. RT-PCR test is the gold standard test for COVID-19, but the RADT can be used in addition to RT-PCR as part of the testing strategies for COVID-19.

## Data Availability Statement

The raw data supporting the conclusions of this article will be made available by the authors, without undue reservation.

## Ethics Statement

The studies involving human participants were reviewed and approved by National COVID-19 Research and Ethics Committee (approval code: CRT-COVID-2020-088). Written informed consent from the participants' legal guardian/next of kin was not required to participate in this study in accordance with the national legislation and the institutional requirements.

## Author Contributions

AA and FM analyzed the data and drafted the manuscript. BA and AlA performed data collection. AA and MA interpreted data and edited the manuscript. SlM edited and revised the manuscript. MA supervised data collection and data analysis, and is the guarantor of this work. All authors reviewed and approved the final version of the manuscript.

## Conflict of Interest

The authors declare that the research was conducted in the absence of any commercial or financial relationships that could be construed as a potential conflict of interest.

## Publisher's Note

All claims expressed in this article are solely those of the authors and do not necessarily represent those of their affiliated organizations, or those of the publisher, the editors and the reviewers. Any product that may be evaluated in this article, or claim that may be made by its manufacturer, is not guaranteed or endorsed by the publisher.
